# Supplementary material for: Avian Influenza Virus and Avian Paramyxoviruses in Wild Waterfowl of the Western Coast of the Caspian Sea (2017–2020)
Source: Viruses. 2024 Apr 12;16(4):598. doi: 10.3390/v16040598 (PMC11054612; doi:10.3390/v16040598)
Supplement: Supplementary file 1 [file viruses-16-00598-s001.zip › viruses-2888386-supplementary.pdf]

Table S1. Isolates of hemagglutinating viruses isolated in 2017-2020.

	Bird species	Virus/subtype	Strain name
1.	Mallard (*Anas platyrhynchos*)	H7N3	A/mallard/Dagestan/1050/2018
2.	Mallard (*Anas platyrhynchos*)	H7N3	A/mallard/Dagestan/1051/2018
3.	Mallard (*Anas platyrhynchos*)	H1N1	A/mallard/Dagestan/1092/2018
4.	Mallard (*Anas platyrhynchos*)	H1N1	A/mallard/Dagestan/1093/2018
5.	Common Pheasant (*Phasianus colchicus*)	APMV	NDV/common pheasant/ Dagestan/Russia/33/2018
6.	Common Teal (*Anas crecca*)	H12N5	A/teal/Dagestan/1017/2018
7.	Gadwall (*Anas strepera*)	H4N6	A/gadwall/Dagestan/156/2017
8.	Common Teal (*Anas crecca*)	APMV-6	APMV6/teal/Dagestan/62/2017
9.	Gadwall (*Anas strepera*)	APMV-4	APMV4/teal/Dagestan/143/2017
10.	Common Teal (*Anas crecca*)	H3N8	A/teal/Dagestan/44/2018
11.	Common Teal (*Anas crecca*)	APMV	NDV/common teal/Dagestan/Russia/111/2017
12.	Common Teal (*Anas crecca*)	H3N8	A/teal/Dagestan/114/2017
13.	Common Teal (*Anas crecca*)	APMV-6	APMV6/teal/Dagestan/130/2017
14.	Common Teal (*Anas crecca*)	APMV	NDV/common teal/Dagestan/Russia/54/2017
15.	Common Teal (*Anas crecca*)	H3N8	A/teal/Dagestan/59/2017
16.	Common Pochard (*Aythya ferina*)	H3N8	A/pochard/Dagestan/92/2017
17.	Common Teal (*Anas crecca*)	H3N8	A/teal/Dagestan/670/2017
18.	Common Teal (*Anas crecca*)	H3N8	A/teal/Dagestan/141/2017
19.	Red-crested Pochard (*Netta rufina*)		
20.	Common Teal (*Anas crecca*)	APMV-4	APMV-4/common teal/Dagestan/Russia/114/2017
21.	Common Teal (*Anas crecca*)	H3N8	A/teal/Dagestan/82/2017
22.	Mallard (*Anas platyrhynchos*)	APMV-4	APMV4/mallard/Dagestan/92d/2018
23.	Northern Pintail (*Anas acuta*)	n.d.	n.d.
24.	Mallard (*Anas platyrhynchos*)	H10N5	A/mallard/Dagestan/8/2018
25.	Common Teal (*Anas crecca*)	APMV-6	APMV-6/common teal/Dagestan/Russia/62/2017
26.	Garganey (*Anas querquedula*)	APMV	APMV-4/garganey/ Dagestan/Russia/143/2017
27.	Garganey (*Anas querquedula*)	H3N8	A/teal/Dagestan/120/2017
28.	Garganey (*Anas querquedula*)	H3N8	A/teal/Dagestan/23/2018
29.	Garganey (*Anas querquedula*)	H8N4	A/teal/Dagestan/57/2018
30.	Mallard (*Anas platyrhynchos*)	APMV-4	APMV4/mallard/Dagestan/1

			35c/2018
31.	Common Teal (*Anas crecca*)	APMV	APMV-4/common teal/Dagestan/Russia/72_1/2017
32.	Coot (*Fulica atra*)	n.d.	n.d.
33.	Tufted Duck (*Aythya fuligula*)	APMV6	APMV6/ Tufted/Dagestan/194/2018
34.	Coot (*Fulica atra*)	n.d.	n.d.
35.	Mallard (*Anas platyrhynchos*)	APMV4	APMV4/mallard/Dagestan/59/2018
36.	Mallard (*Anas platyrhynchos*)	APMV	APMV-6/mallard/ Dagestan/194d/Russia/2020
37.	Mallard (*Anas platyrhynchos*)	AIV	n.d.
38.	Garganey (*Anas querquedula*)	n.d.	n.d.
39.	Garganey (*Anas querquedula*)	AIV	n.d.
40.	Coot (*Fulica atra*)	AIV	n.d.
41.	Coot (*Fulica atra*)	n.d.	n.d.
42.	Coot (*Fulica atra*)	AIV	n.d.
43.	Common Teal (*Anas crecca*)	H4N6	A/teal/Dagestan/34/2019

Note: n.d. – not determined

Analysis of the PB2 segment

In the PB2 segment, all strains studied belong to the Eurasian genetic line of avian influenza viruses. The sequences form two main phylogenetic clades (Fig. S1).

The PB2 segments of strains A/pochard/Dagestan/92/2017 and A/teal/Dagestan/120/2017 form a subclade closely related to the strains from Asia, more specifically East, South, and North Asia. Strain A/gadwall/Dagestan/156/2017 falls into a separate phylogenetic subclade of sequences isolated in different parts of Asia, Africa, and Eastern Europe.

The PB2 of the other strains from Dagestan are similar to strains widely distributed throughout Eurasia, as well as those isolated in South Asia and northeast Africa. In particular, the strain A/teal/Dagestan/82/2017 is similar in segment PB2 to strains from Siberia and Mongolia.

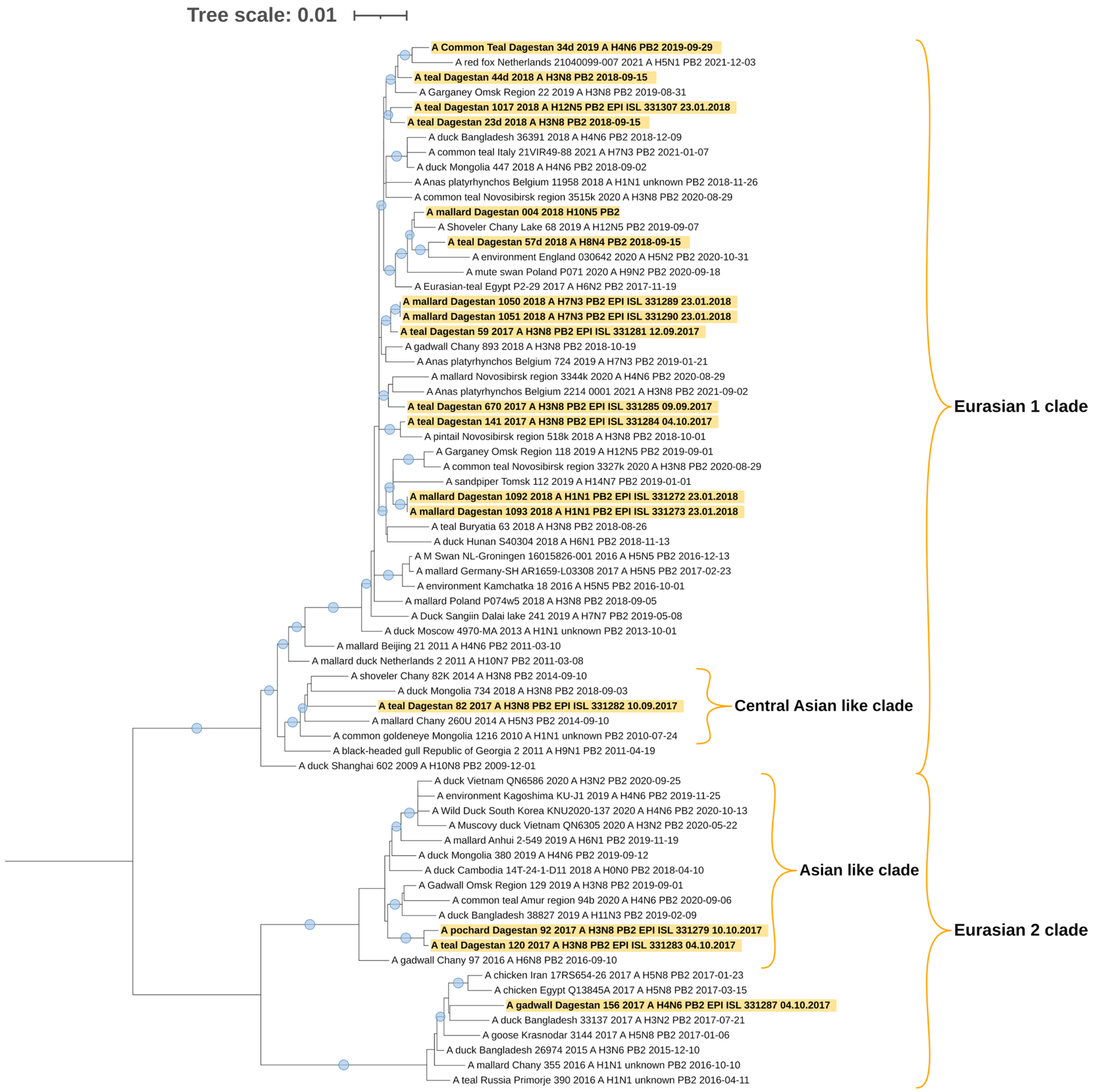

Figure S1. Maximum likelihood phylogenetic tree of the PB2 genome segment of avian influenza virus strains isolated in the west Caspian region (2017-2019). The blue circle symbol denotes branches with values SH-aLRT > 80% and UFboot > 95%.

Analysis of the PB1 segment

In the PB1 segment, all strains studied belonged to the Eurasian genetic line of avian influenza viruses. The sequences form two main phylogenetic clades (Fig. S2).

In the first phylogenetic clade, segments PB1 of strains A/pochard/Dagestan/92/2017, A/teal/Dagestan/141/2017, and A/teal/Dagestan/23d/2018 can be distinguished and are similar to PB1 strains isolated mainly in parts of Europe (Czech Republic, Poland, Hungary, Germany, and Georgia). A couple of strains with a similar PB1 segment were also found in Siberia and Bangladesh. The remaining strains from Dagestan in this clade are fairly evenly distributed and are related to strains from different parts of Eurasia.

The second phylogenetic clade is formed by the PB1 strains A/mallard/Dagestan/004/2018, A/teal/Dagestan/82/2017, A/teal/Dagestan/1017/2018, A/CommonTeal/Dagestan/34d/2019, and A/teal/Dagestan/44d/2018, as well as strains

isolated in West Asia, South Asia, East Asia, North Asia, northeast Africa, and East and West Europe.

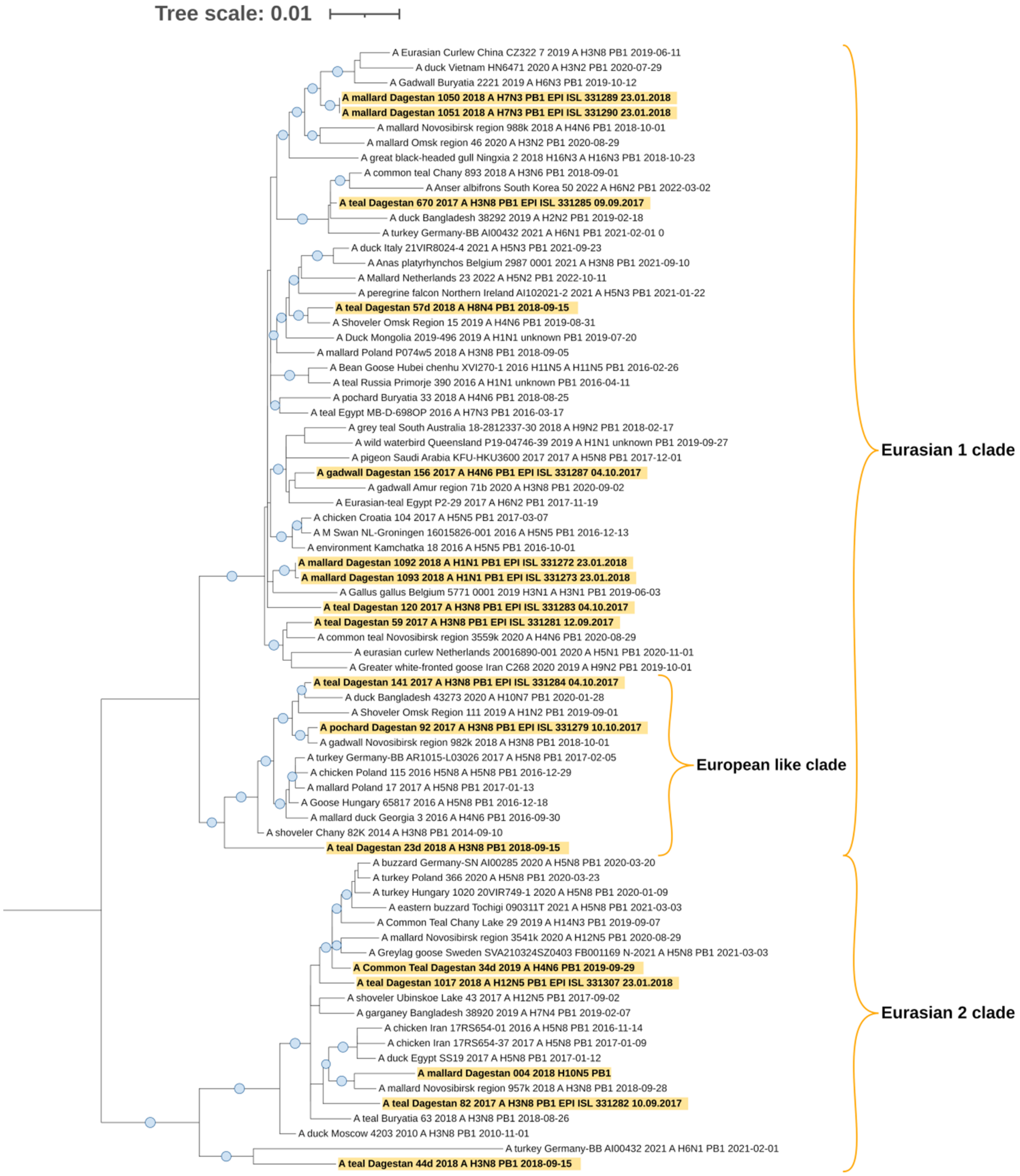

Figure S2. Phylogenetic analysis of the PB1 genome segment of avian influenza virus strains isolated in the west Caspian region (2017-2019). The blue circle symbol denotes branches with values SH-aLRT > 80% and UFboot > 95%.

Analysis of the PA segment

All strains studied of the PA segment belong to the Eurasian genetic line of avian influenza viruses and form two main phylogenetic clades on the dendrogram, which, in turn, are divided into subclades (Fig. S3).

The PA sequences of strain A/teal/Dagestan/57d/2018 form a subclade with strains isolated mainly in Asia (East, North, and South). The strains A/mallard/Dagestan/1092/2018, A/mallard/Dagestan/1093/2018, and A/teal/Dagestan/44d/2018 are similar to influenza virus variants isolated in Europe.

The other PA sequences of strains from Dagestan, which are grouped into two subclades within one clade, show a similarity to various Eurasian strains.

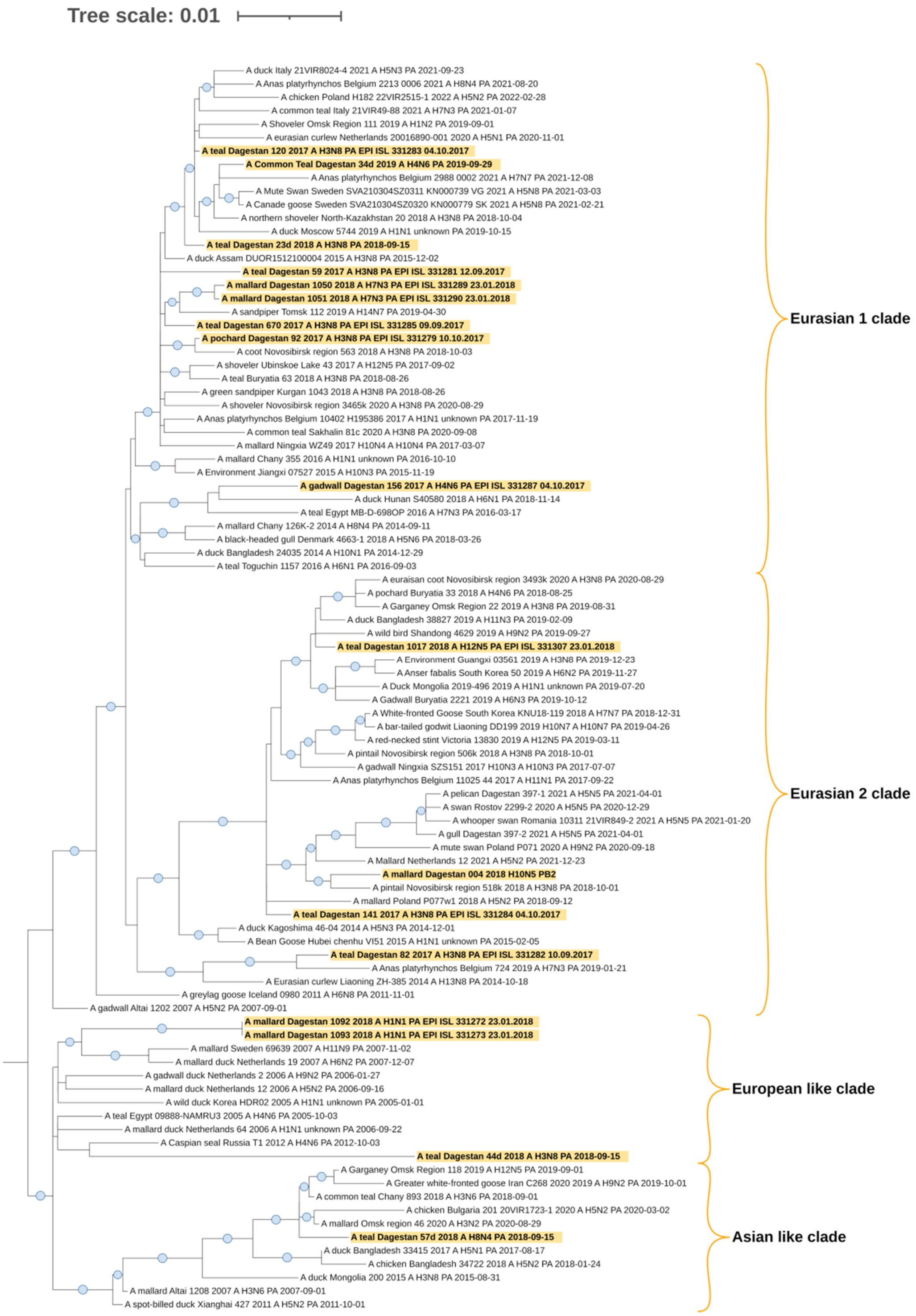

Figure S3. Phylogenetic analysis of the PA genome segment of avian influenza virus strains isolated in the west Caspian region (2017-2019). The blue circle symbol indicates branches with values of SH-aLRT > 80% and UFboot > 95%.

Analysis of the NP segment

The sequences of NP segments of all the strains studied belong to the Eurasian genetic lineage of avian influenza viruses and form two main phylogenetic clades and several subclades within these clades (Fig. S4).

In the first phylogenetic clade, strains A/teal/Dagestan/120/2017, A/teal/Dagestan/1017/2018, A/teal/Dagestan/23d/2018, and A/CommonTeal/Dagestan/34d/2019 form a subclade with strains obtained mainly from East and West Europe, as well as East and North Asia. Strains

A/mallard/Dagestan/004/2018, A/mallard_Dagestan/1050/2018, A/mallard/Dagestan/1051/2018, A/gadwall/Dagestan/156/2017, A/teal/Dagestan/57d/2018, and A/teal/Dagestan/59/2017 form a subclade together with strains from different parts of Asia, as well as with strains from northeast Africa.

The second phylogenetic clade is formed by sequences of NP strains from Dagestan, as well as strains isolated in Europe, West Asia, East Asia, and the Far East. At the same time, the sequences of the NP segment of strains A/mallard/Dagestan/1092/2018, A/mallard/Dagestan/1093/2018, A/pochard/Dagestan/92/2017, A/teal/Dagestan/141/2017, and A/teal/ Dagestan/44d/2018 are similar to NPs of various subtypes isolated in Europe and West Asia, as well as to NPs of the highly pathogenic strain of the H5N8 subtype isolated in Korea. The sequence of the NP segment of strain A/teal/Dagestan/82/2017 belongs to another phylogenetic subclade and is close to the NP strains of avian influenza virus isolated, with the exception of the sample from Georgia, in North Asia, East Asia, and South East Asia.

The sequence of NP strain A/teal/Dagestan/670/2017 and similar NP variants of the influenza virus isolated in the East Asian region, as well as in Europe and Australia, form a separate subclade.

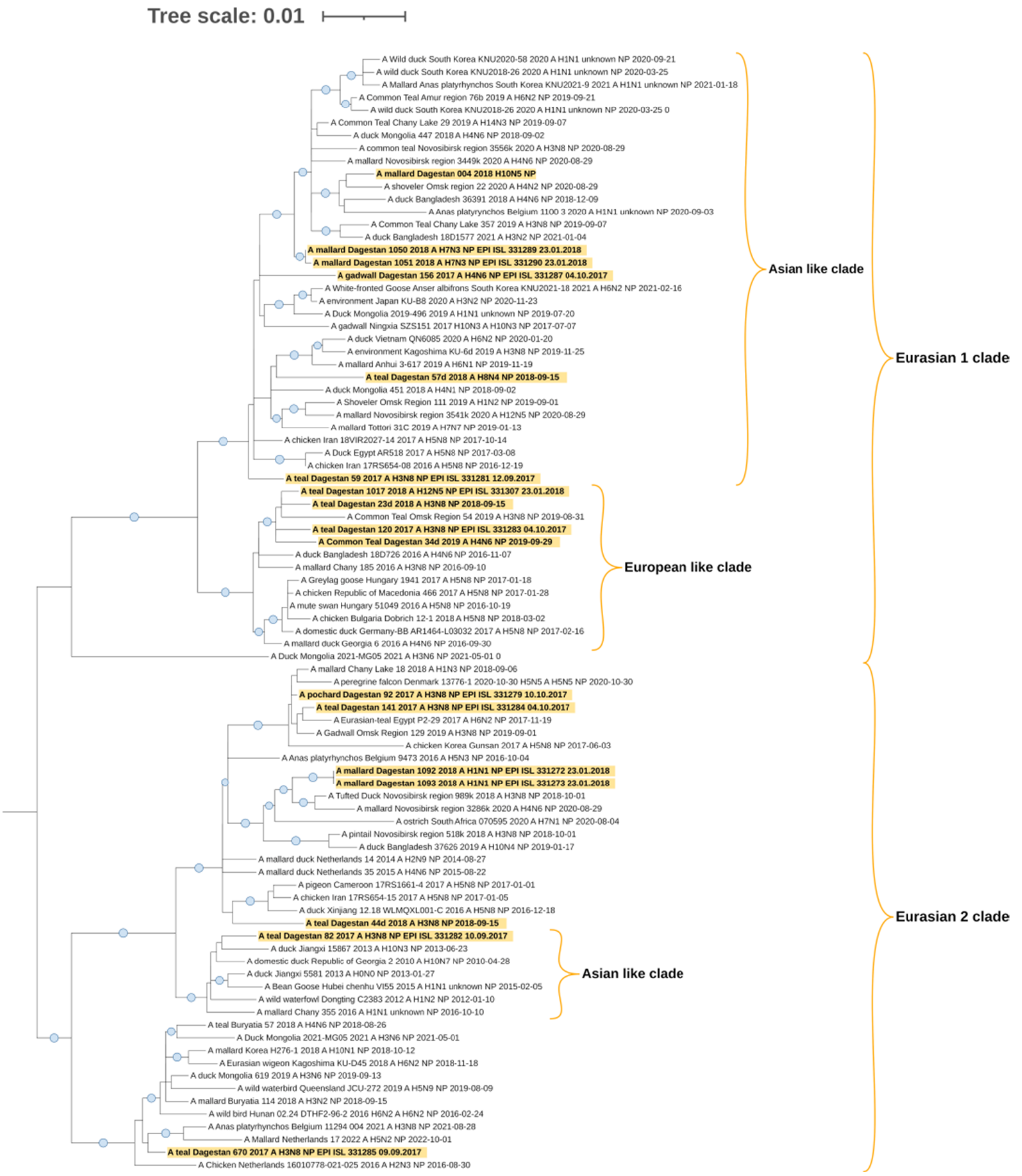

Figure S4. Phylogenetic analysis of the genome segment of NP strains of avian influenza virus isolated in the west Caspian region (2017-2019). The blue circle symbol denotes branches with values SH-aLRT > 80% and UFboot > 95%.

Analysis of the MP segment

The MP segments of all studied strains isolated in Dagestan belong to the Eurasian genetic line of avian influenza viruses. On the phylogenetic dendrogram they are distributed quite evenly (Fig. S5). Strains A/Common_Teal/Dagestan/34d, A/mallard/Dagestan/1050/2018, A/mallard/Dagestan/1051/2018, A/teal/Dagestan/1017/2018, A/teal/Dagestan/44d/2018, A /gadwall/Dagestan/156/2017, and A/teal/Dagestan/59/2017 show similarity to strains from different parts of Eurasia, as well as to a strain from South Africa. The nucleotide sequences of MP strains A/teal/Dagestan/82/2017 and A/teal/Dagestan/23d/2018 are similar to MP strains isolated mainly in North and East Asia (including the Far East).

MP strains A/mallard/Dagestan/004/2018, A/mallard/Dagestan/1092/2018, A/mallard/Dagestan/1093/2018, A/pochard/Dagestan/92/2017, and A/teal/Dagestan/670/2017 are similar to MR strains isolated in North Asia, South Asia, northeast Africa, West and East Europe, and Australia. The A/teal/Dagestan/141/2017 strain is also similar in the MR segment to variants of the influenza virus from East Asia (including the Far East), Central Asia, and South Asia. Strains A/teal/Dagestan/120/2017 and A/teal/Dagestan/57d/2018 are most similar to strains from Europe (Netherlands, Denmark).

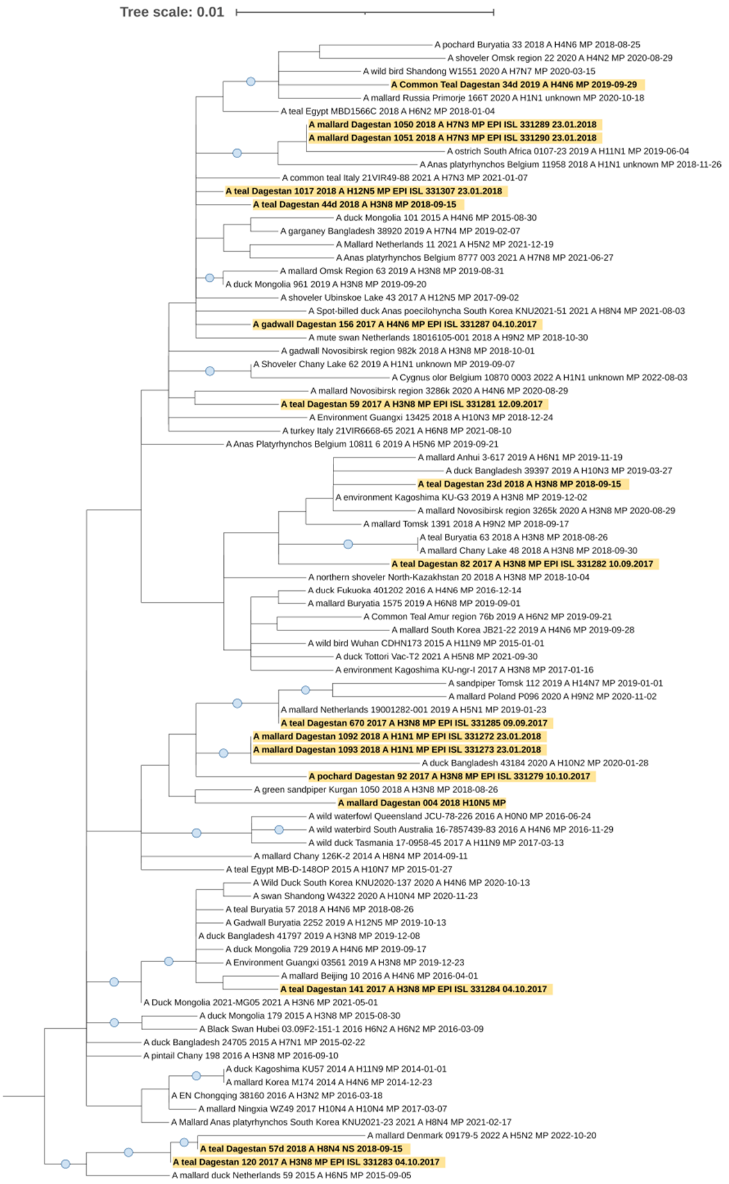

Figure S5. Phylogenetic analysis of the MP genome segment of avian influenza virus strains isolated during the project in the west Caspian region (2017-2019). The blue circle symbol denotes branches with values SH-aLRT > 80% and UFboot > 95%.

Analysis of the NS segment

The NS segments of all influenza virus strains studied from Dagestan belong to the Eurasian genetic lineage. In addition, according to the phylogenetic dendrogram, they are divided into two radically different genetic clades—the so-called "allele A" and "allele B"—of the NS influenza virus (Fig. S6). Those influenza viruses which contain the most genetically diverse NS segments are found in the Caspian Sea region. Further, within these clades, NS segments are divided into subclades.

The NS of the A/mallard/Dagestan/1092/2018, A/mallard/Dagestan/1093/2018, A/mallard/Dagestan/004/2018, and A/teal/Dagestan/44d/2018 strains included in the A allele are similar to the NS of influenza viruses isolated from birds in Europe, Western Asia, South Asia, Southeast Asia, and Northeast Africa. Strains of the second subclade of allele A (A/gadwall/Dagestan/156/2017, A/mallard/Dagestan/1050/2018, and A/mallard/Dagestan/1051/2018) are similar to variants of the avian influenza virus from Europe, West Asia, North Asia, and East Asia.

The NS B allele is also heterogenic. NS sequences A/teal/Dagestan/141/2017, A/teal/Dagestan/1017/2018, A/teal/Dagestan/57d/2018, A/CommonTeal/Dagestan/34d/2019, and A/teal/Dagestan/23d/ 2018 are phylogenetically close to NS strains from West and North Europe, Central Asia, East Asia, and South Asia (including those isolated much earlier, in 1999–2009).

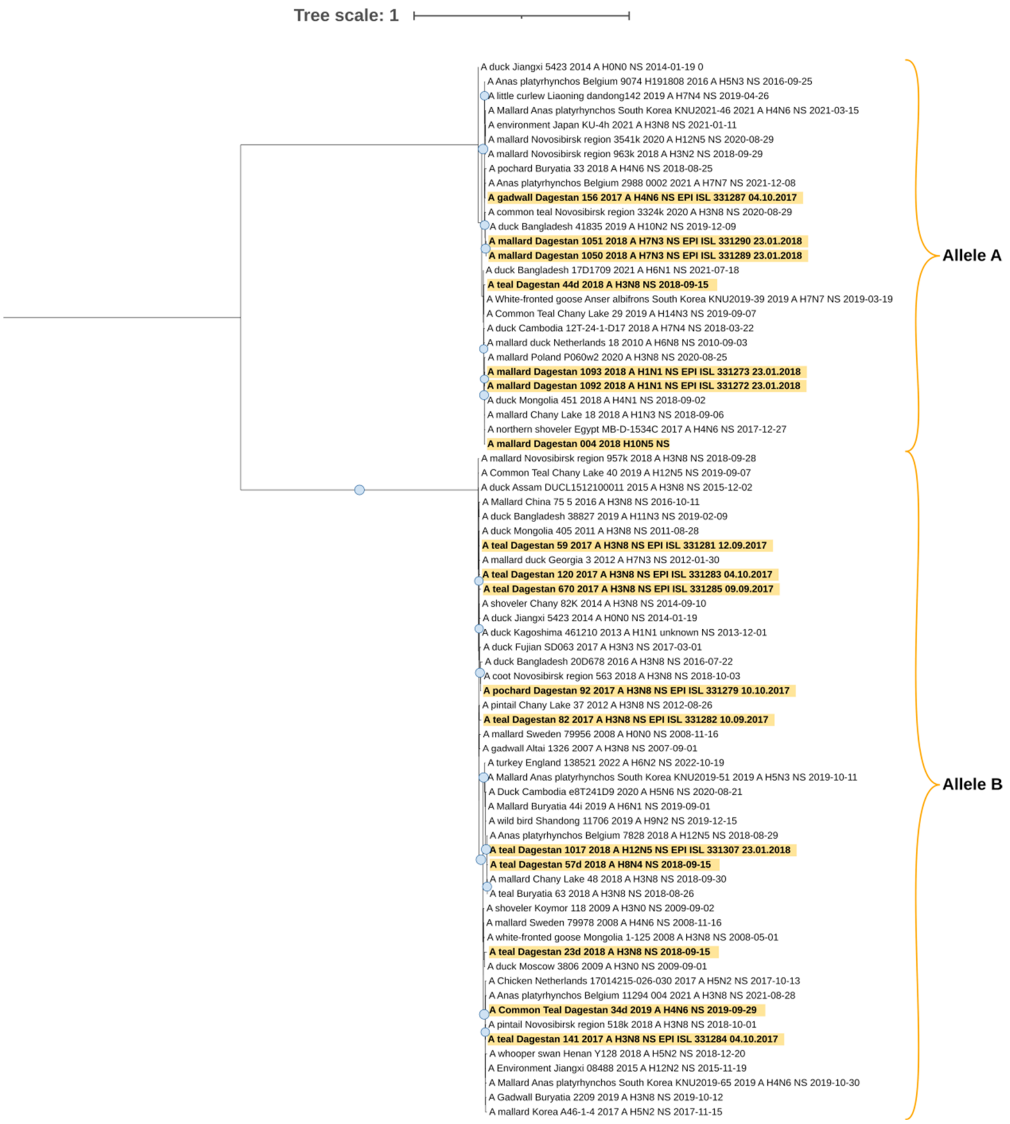

Figure S6. Phylogenetic analysis of the genome segment NS of avian influenza virus strains isolated in the west Caspian region (2017-2019). The blue circle symbol denotes branches with values SH-aLRT > 80% and UFboot > 95%.
